# Gene expression of adipokines in placentas of cows from Northeastern Brazil with and without hyperketonemia and their interactions with metabolic biomarkers

**DOI:** 10.1007/s11250-026-05288-z

**Published:** 2026-08-10

**Authors:** Ângela Imperiano da Conceição, João Victor José de Barros Dantas, Carla Lopes de Mendonça, Jobson Filipe de Paula Cajueiro, Rodolfo José Cavalcanti Souto, Licarion Pinto, Daniel Nunes de Araújo Gonçalves, Paulo Roberto Eleutério de Souza, José Augusto Bastos Afonso, Pierre Castro Soares

**Affiliations:** 1 Postgraduate Program in Veterinary Medicine, Federal Rural University of Pernambuco, Recife, Pernambuco Brazil; 2 Department of Biology, Federal Rural University of Pernambuco, Recife, Pernambuco Brazil; 3 Federal Rural University of Pernambuco, Cattle Clinic, Garanhuns, Pernambuco Brazil; 4 Department of Analytical Chemistry, Institute of Chemistry, State University of Rio de Janeiro, Rio de Janeiro, Brazil; 5 Department of Veterinary Medicine, Federal Rural University of Pernambuco, Recife, Pernambuco Brazil

**Keywords:** Ketosis, Leptin, Adiponectin, Cattle, Multivariate analysis

## Abstract

The objective was to analyze the mRNA expression of leptin, leptin receptor (ObRb), adiponectin, adiponectin receptor (AdipoR1), and resistin in the placental tissue of cows with and without hyperketonemia, relating it to their metabolic profile. Samples were collected from 135 cows during birth, divided into two groups: G1: without hyperketonemia (*n* = 120) and G2: with hyperketonemia (*n* = 15). Biochemical and hormonal indicators and insulin sensitivity were evaluated. Placental fragments were analyzed by qPCR. The variables were tested for normality and Pearson’s correlation coefficient was applied. The significance level was 5%. Cows with hyperketonemia showed increased concentrations of NEFA (*p* = 0.0002), urea (*p* = 0.0302), higher expressions of leptin (*p* = 0.0174) and ObRb (*p* < 0.0001), while healthy cows showed higher expression of AdipoR1 (*p* < 0.0001) and resistin (*p* = 0.0435). Glucose concentrations were lower in G2 (*p* = 0.0132), as were the percentages of RQUICKI_βHB_ (*p* < 0.0001). Among the varying degrees of correlations between the variables, the following stand out: positive correlation of leptin expression with β-Hydroxybutyrate and ObRb expression; positive correlation of ObRb expression with β-hydroxybutyrate, NEFA, GGT and AST, and negative correlation with glucose; positive correlation of AdipoR1 expression with glucose, RQUICK and RQUICK_βHB_, and negative correlation with β-Hydroxybutyrate, NEFA and GGT. Gene expression analysis of adipokines in the placenta is a promising tool to understand their role in metabolic regulation in cows, especially in the dynamics of hyperketonemia. It is concluded that the placental production of these hormones has an important contribution to metabolic conditions in the transition period, bringing implications for the physiology, health and productivity of females.

## Introduction

Hyperketonemia, a condition widely disseminated in cattle herds around the world (Loiklung et al. 2022), occurs as a result of alterations in energy metabolism, originating from the accentuated negative energy balance (NEB) that affects females in the final third of pregnancy and/or at the beginning of lactation (Duffield et al. 2009; McArt et al. 2013). However, it is important to highlight that this condition does not necessarily affect all cows clinically, as the intensity of the clinical impact changes according to the individual’s ability to adapt metabolically to support NEB and metabolize and tolerate ketone bodies. Cows with serum β-hydroxybutyrate (BHB) concentrations ≥ 1.2 mmol/L are considered hyperketonemic, encompassing both subclinical and clinical presentations of this metabolic disorder (Duffield et al. 2009; Gordon et al. 2013; McArt et al. 2013; Tatone et al. 2016; Soares et al. 2019). This imbalance results in significant economic losses, reflected in treatment costs and reduced milk production, in addition to increasing the risk of developing other commonly associated diseases (LeBlanc 2010; Gordon et al. 2013; Soares et al. 2019; Tatone et al. 2017; Loiklung et al. 2022).

Despite the discovery of these mechanisms for the development of hyperketonemia, it is still uncertain whether all the pathways that contribute to the onset of these diseases are fully established. It is known that other hormones play a role in energy metabolism, for example adipokines, such as leptin, adiponectin, and resistin, whose roles are fundamental in modulating systems and homeostatic control of energy in the body (Reverchon et al. 2014; Kasimanickam 2016; Roh et al. 2016; Shen et al. 2020a; Guadix et al. 2023). Therefore, understanding the physiological roles of adipokines and their interrelationships with different biomarkers of metabolism in ruminants is crucial to understanding the dynamics of metabolic homeostasis and the physiological performance of these females, as well as improving animal health and productivity, since they are intrinsically related to states of metabolic regulation, energy balance, glucose and lipid metabolism, oxidative stress, reproductive and immune systems, and endocrine responses, affecting insulin sensitivity and metabolic syndromes (Godoy-Matos et al. 2014; Roh et al. 2016; Shen et al. 2020a, b).

Physiological fluctuations resulting from changes in diet, gestation, and lactation can modify the energy metabolism of ruminants, reflecting in adipokine concentrations, some of which are produced in high concentrations in the placenta. These molecules exert multifaceted influences on placental tissue, fetal development, and maternal energy metabolism, with evidence suggesting the participation of these adipokines in the pathogenesis of hyperketosis, through the interaction between body fat, energy expenditure, insulin sensitivity, glucose uptake by tissues, and regulation of fatty acid oxidation (Guadix et al. 2023; Kasimanickam 2016; Pérez-Pérez et al. 2018; Acquarone et al. 2019; Shen et al. 2020a).

Although circulating adipokines are already well established as biomarkers of metabolic status during the transition period, the role of the placenta as an active source and modulator of these molecules in the context of negative energy balance and hyperketonemia remains poorly understood (Kasimanickam 2016; Roh et al. 2016; Shen et al. 2020a, b). In this context, the present study advances the field by investigating the local gene expression of placental adipokines and their integration with the systemic metabolic profile, demonstrating that the placenta should be interpreted not only as a target organ but also as a functional component in maternal–fetal metabolic regulation. By correlating the expression of leptin, adiponectin, resistin, and their receptors with metabolic biomarkers, the findings broaden the understanding of the pathophysiological mechanisms underlying hyperketonemia and suggest that local alterations may reflect relevant systemic adaptations. From an applied perspective, these results indicate that placental adipokine expression may, in the future, contribute to improving metabolic monitoring during the peripartum period, as well as support more targeted nutritional and reproductive strategies, with potential impacts on the prevention of metabolic disorders and the optimization of productive and reproductive performance in dairy herds.

Given this context, the present study aimed to investigate the expression of mRNA for leptin, adiponectin, their receptors and resistin, in the placental tissue of cows with and without hyperketonemia, as well as to evaluate the interaction of these adipokines with the energetic, protein, mineral, enzymatic, and hormonal metabolic profile of these females.

## Materials and methods

### Animals

The study included pregnant cows (*n* = 135), coming from herds in the state of Pernambuco, Brazil, sent for clinical-obstetric care (eutocic birth, obstetric maneuver, or cesarean section) at the Garanhuns Bovine Clinic/UFRPE, which were subjected to clinical examination (Dirksen et al. 1993), to define the obstetric diagnosis or other problems. Of these females, 79 underwent cesarean section surgery, 42 underwent obstetric maneuvers, and 14 had eutocic births. The cows were aged between one and ten years, with a body score ranging from 2.5 to 4 (Edmonson et al. 1989), and belonged to the dairy breeds Girolando, Holstein, Jersey, Gir, and Brown Swiss, as well as crossbreeds. The cows were raised predominantly in a semi-intensive system, receiving diets containing varied forage, mostly composed of elements such as grass (different varieties/native pasture), palm, sugar cane bagasse, corn silage and cassava peel, in addition to concentrated feed and mineral salt.

Samples were collected exclusively at the time of birth. Subsequently, the females were categorized into groups, according to the inclusion indicators of the study: G1: Group without hyperketonemia/Control (CG/*n* = 120), animals without clinical alterations presenting serum concentrations of β-hydroxybutyrate < 1.2mmol/L; and G2: Group with hyperketonemia subclinical (HyperketonemiaG/*n* = 15), animals presenting serum concentrations of β-hydroxybutyrate ≥ 1.2mmol/L (Kaneko et al. 2008; Gordon et al. 2013; McArt et al. 2013; Loiklung et al. 2022).

## Sample collection and storage

Blood samples were collected by jugular venipuncture, using a tube for a vacuum collection system (25mmx0.8 mm) and siliconized vacutainer^→^ tubes, without anticoagulant and with sodium fluoride associated with ethylenediaminetetraacetic acid (EDTA), to obtain serum and plasma, respectively. These were subjected to centrifugation for 15 min. at 3600 g, aliquoted, and packaged in a plastic microtube and stored at a temperature of -20 °C for subsequent biochemical analyses.

Placental tissue samples were collected after spontaneous expulsion or during obstetric procedures (eutocic delivery, obstetric maneuver, or cesarean section), with portions close to the umbilical cord being selected (Shein et al., 2020a). Subsequently, the tissues were washed with DEPC water^1^(RNase-free), stored in 2mL cryovials containing 300µL of Trizol^→^ and kept at -80 °C until total RNA extraction.

## Blood biochemistry

The biochemical variables analyzed were β-hydroxybutyrate^2^, non-esterified fatty acids/NEFA^3^, glucose^4^, total protein^5^, albumin^6^, urea^7^, creatinine^8^, serum activity of gamma-glutamyltransferase/GGT^9^, serum activity of aspartate aminotransferase/AST^10^, chlorides^11^, and phosphorus^12^, using a semi-automatic biochemical analyzer (Kaneko et al. 2008).

Serum concentrations of insulin^13^ and cortisol^14^ were determined by chemiluminescent immunoassay. Serum concentrations of ionized calcium (Ca^++^), sodium (Na^+^), and potassium (K^+^) were determined using an electrolyte analyzer. To obtain the difference in strong ions, the following equation was used: DIF = ([Na]Serum + [K]Serum) – [Cl]Serum) (Constable and Stämpfli 2005; Masevicius et al. 2013).

## Insulin sensitivity assessment

Insulin sensitivity was assessed using the Revised Quantitative Insulin Sensitivity Check Index (RQUICKI) and RQUICKI-_BHB_, which uses the concentrations of glucose, insulin, NEFA, and BHB according to the following equations: RQUICKI = 1/[log10 (glucose, mg dL-1) + log10 (insulin, µU mL-1) + log10 (NEFA, mmol L-1)]; and RQUICKI-BHB = 1/[log10 (glucose, mg dL-1) + log10 (insulin, µU mL-1) + log10 (NEFA, mmol L-1) + log10 (BHB, mmol L-1)] (Perseghin et al. 2001; Holtenius and Holtenius 2007; Djoković et al. 2017).

## Adipokine mRNA gene expression

Of the 135 placenta samples collected from cows, 38 were processed and analyzed, 23 from cows in the control group and 15 from the hyperketonemia group. This selection was based on the quantity/concentration and quality of mRNA extracted from each sample, which allowed for the completion of all processing steps, as well as the completeness of the metabolic data. Total RNA extraction was performed using the Trizol method, according to the manufacturer’s instructions. Subsequently, the RNA was quantified by spectrophotometry, and its integrity was assessed using SYBR Green II in 1% agarose gel. The RNA samples were stored at − 80 °C until complementary DNA (cDNA) preparation. cDNA was synthesized from 1 µg of total RNA, using the commercial kit according to the manufacturer’s recommendations, and stored at -20 °C.

Real-time PCR analyses were performed using the Rotor-GENE Q, with a total volume of 20µL per reaction, with 2µL of diluted cDNA (1 cDNA: 9 H2O DEPC), 0.8µL of forward and reverse primers (100µM), assay specific, 10µL of the commercial qPCR kit, and 6.4µL of water, with the following settings: 94 °C for 15 min, followed by 40 cycles of 94 °C for 30 s, primer-specific annealing temperature for 30 s, and 72 °C for 45 s. Reactions were performed in duplicate for each sample, together with the negative control. The defined endogenous control was RNA polymerase II (Duarte et al. 2014). The primers to verify the expression of leptin, leptin receptor (ObRb), adiponectin, adiponectin receptor (AdipoR1), and resistin were designed based on the sequence available in GenBanK (Table 1).

**Table 1:** Sequences of specific primers for mRNA expression of leptin, leptin receptor (ObRb), adiponectin, adiponectin receptor (AdipoR1), resistin, and RNA polymerase II (RNApolII)

Product	Primer sequence		Size (pb)	Temperature pairing
Leptin		F: GACATCTCACACACGCAG R: GAGGTTCTCCAGGTCATT	183	50 °C
ObRb		F: ACCACACCTTCCGTTCTCAG R: GGGACAACACTCTTGACTC	164	45 °C
Adiponectin		F: CTGGAGAGAAGGGAGAGAAAAG R: TGGGTACATTGGGAACAGTG	204	48 °C
AdipoR1		F: GGCTCTACTACTCCTTCTAC R: ACACCCCTGCTCTTGTCTG	118	47 °C
Resistin		F: CTGCCCTTCAGGCTTCGCCG R: GGCAGTGGCACGTGGTCTCA	300	50 °C
RNApolII		F: GAAGGGGGAGAGACAAACTG R: GGGAGGAAGAAGAAAAAGGG	86	49 °C

Relative quantification of ΔΔCt was performed according to Livak and Schmittgen (2001). The difference between the mean Ct of the gene of interest and the mean Ct of the endogenous gene (ΔCT) was calculated for normalization. The relative expression of each gene was calculated using the ΔΔC formula, and the result for gene expression was determined by a dimensionless value using the formula 2^−ΔΔCt^.

### Statistical analysis

First, the data were subjected to the normality test (Kolmogorov-Smirnov) and homoscedasticity test (Levene), based on assumptions about the nature of the data, whether they followed an approximately normal distribution (Gaussian curve), and whether the data from the groups presented constant variance, respectively, ensuring that the insights obtained were reliable and replicable. For this model, a significance level of 5% probability was considered.

For the total set of variables, those that required transformation (logarithmic or radical) were BHB, AST, GGT, Cai, Insulin, Cortisol, HOMA-IR, QUICKI, RQUICKI and RQUICKIBHB, ExpreLept, ExpreObRb, ExpreAdipo, ExpreAdipoR1 and ExpreResist. After a new procedure of analyzing the assumptions of normality and homoscedasticity, it was found that the variables that remained without a Gaussian normal distribution were AST, GGT, Cortisol, HOMA-IR, and QUICKI, and these were subjected to a non-parametric inferential statistical model test. For the other variables with a Gaussian distribution, parametric tests were applied.

For the set of variables with a parametric profile, an analysis of variance (F-test) was performed, followed by a comparison of sample means using the Student-Newman-Keuls test (SNK test). For the set of variables with a non-parametric profile, the data were subjected to the Mann-Whitney U test. The variables studied with a parametric profile are expressed as means and standard error, while those with a non-parametric profile are expressed as medians and percentiles (P25 and P75). The statistical package Statistical Analysis System (SAS, 2000) was used for the analyses, with the SAS PROC MIXED procedure.

Additionally, a correlation analysis was performed between pairs of studied variables using Pearson’s correlation coefficient. The significance obtained in the linear correlation was evaluated according to Little and Hills (1978), establishing a high-intensity correlation between the variables when *r* > 0.60; medium intensity when 0.30 > *r* < 0.60; and low intensity when *r* < 0.30. The significance level obtained for all correlations was *p* < 0.05. For the associative analysis (X and Y correlation), no adjustments for multiple comparisons were applied. We chose to treat this analysis as exploratory, since the objective was to identify variables applicable to correlation analysis for future studies, and not to confirm final hypotheses. The interpretation of correlations (especially those with a threshold p-value) prioritized the effect size over the applied p-value, so as not to interpret them as “trends” or “near significance,” but rather as a result that does not reject the null hypothesis based on the 5% probability level applied in the statistical protocol.

## Results

### Dynamics of metabolites of the biochemical energetic profile in cows with and without hyperketonemia

The serum concentrations of β-hydroxybutyrate and NEFA were marked in the group of animals with hyperketonemia in relation to the control (*p* < 0.0001 and 0.0002, respectively). Mean plasma glucose values were significantly different between the groups, with higher concentrations found in control group cows compared to cows with hyperketonemia (Table 2).

**Table 2:** Means, standard deviations, and significance levels of metabolites in the biochemical energetic profile of cows with and without hyperketonemia, at the time of calving

Variables	Groups		“*p*” level	References values
	Control (*n* = 120)	Hyperketonemia (*n* = 15)		
**Energy** ^#^				
β-hidroxibutirato (mmol/L)	0,71 ± 0,06 ^B^	2,22 ± 0,14 ^A^	<,0001	> 1,2 ^1^
AGNE (mmol/L)	1,14 ± 0,12 ^B^	2,19 ± 0,19 ^A^	0,0002	> 0,4 ^1^
Glucose (mmol/L)	5,44 ± 0,23 ^A^	4,30 ± 0,24 ^B^	0,0132	2,5 − 4,16 ^2^

* Different letters on it indicate differences at the 5% probability level.

# Means and standard error.

1: Gordon et al. (2013); McArt et al. (2013); Loiklung et al. (2022); 2: Kaneko et al. (2008).

### Dynamics of protein profile metabolites, enzymatic activity, and electrolytes in cows with and without hyperketonemia

For the set of variables of the protein profile, enzymatic activity, and electrolytes, only the serum concentration of urea and the enzymatic activity of GGT presented variations between groups, with higher means recorded for cows with hyperketonemia compared to those in the control group. The other variables were not influenced by the hyperketonemia condition (Table 3).

### Dynamics of hormones in cows with and without hyperketonemia

There was no influence of the hyperketonemia condition on serum insulin and cortisol concentrations (Table 4).

### Dynamics of substitutive indices of insulin resistance in cows with and without hyperketonemia

Regarding surrogate indices for insulin resistance, there was no variation between groups for the HOMA-IR, QUICKI, and RQUICKI indices, however significant variations were observed for the RQUICKI_βHB_ index, in which lower percentages were recorded in cows with hyperketonemia in relation to the control group (Table 5).

**Table 3:** Means, standard deviations, medians, percentiles (P_25_ and P_75_), and significance levels of blood concentration of metabolites of the protein profile, enzymatic activity, and electrolytes of cows with and without hyperketonemia, at the time of calving

Variables	Groups		“*p*” level	Reference values ^1^
	Control (*n* = 120)	Hyperketonemia (*n* = 15)		
**Protein** ^#^				
Creatinine (µmol/L)	121,95 ± 5,28	119,10 ± 6,96	0,7848	88,42–176,84
Urea (mmol/L)	4,76 ± 0,24 ^B^	5,93 ± 0,31^A^	0,0302	7,13 − 10,69
Total Protein (g/L)	73,28 ± 1,20	73,97 ± 1,52	0,7990	67,4–74,60
Albumin (g/L)	30,98 ± 0,90	30,45 ± 0,73	0,7532	30,3–35,50
Globulin (g/L)	42,30 ± 1,37	43,52 ± 1,75	0,6789	30–34,80
A: G^##^	0,78 ± 0,04	0,73 ± 0,04	0,5596	0,84 − 0,94
**Enzymatic Activity** ^**#**^				
AST (U/L)	89,05(62,86;141,40)	104,80(62,86;324,80)	0,0933	78–132
GGT (U/L)	22,95(15,30;30,60) ^B^	38,25(15,30;61,20) ^A^	0,0100	6,1–17,40
**Electrolytes** ^**#**^				
Ionic Ca (mmol/L)	0,99 ± 0,02	1,05 ± 0,02	0,0670	1,0–1,4
P (mmol/L)	1,51 ± 0,08	1,51 ± 0,10	0,9684	1,43 − 1,66
Na (mEq/L)	141,70 ± 0,66	142,53 ± 0,68	0,4896	132–152
K (mEq/L)	4,17 ± 0,08	4,10 ± 0,11	0,6895	3,9 − 5,8
Cl (mEq/L)	99,79 ± 0,72	101,06 ± 0,74	0,3673	97–111
DIF (mmol/L)	46,10 ± 0,89	45,63 ± 0,69	0,7779	

*Different letters indicate differences at the 5% probability level. 1: Kaneko et al. (2008).

# Means and standard error. ## Medians and percentiles P25 and P75.

**Table 4:** Means, standard deviations, median, percentiles (P_25_ and P_75_), and significance levels of the hormonal profile of cows with and without hyperketonemia, at the time of calving

Variables	Groups		“*p*” level	Reference values ^1^
	Control (*n* = 120)	Hyperketonemia (*n* = 15)		
Insulin (µIU/mL)^#^	13,00 ± 2,14	6,98 ± 1,28	0,1403	0–5
Cortisol (nmol/L)^##^	137,67 (8,28;299,35)	126,64 (36,14;484,76)	0,4881	15–19

1: Kaneko et al. (2008).

# Means and standard error. ## Medians and percentiles P25 and P75.

**Table 5:** Means, standard deviations, median, percentiles (P_25_ and P_75_), and significance levels of the substitutive indices of insulin resistance of cows with and without hyperketonemia at the time of calving

Tests	Groups		“*p*” level
	Control (*n* = 120)	Hyperketonemia (*n* = 15)	
HOMA-IR (%)^##^	0,30 (0,02;2,30)	0,17 (0,00;0,41)	0,0655
QUICKI (%)^##^	0,48 (0,34;1,02)	0,54 (0,45;24,16)	0,0750
RQUICKI (%)^#^	0,34 ± 0,02	0,29 ± 0,02	0,1071
RQUICK_βHB_ (%)^#^	0,28 ± 0,01^A^	0,18 ± 0,01^B^	<,0001

*Different letters indicate differences at the 5% probability level.

# Means and standard error. ## Medians and percentiles P25 and P75.

### Placental mRNA expression of leptin and other adipokines in cows with and without hyperketonemia

Regarding placental mRNA expressions for leptin and other adipokines in cows, there was no variation between groups for adiponectin expression, but significant variations were observed for the gene expression of leptin, leptin receptor (ObRb), adiponectin receptor (AdipoR1), and resistin, identifying higher means for leptin and ObRb expression in cows with hyperketonemia, and lower means for placental adipoR1 and resistin expressions in cows with hyperketonemia (Table 6).

**Table 6:** Means, standard error of the mean, and significance levels of placental adipokine mRNA expression in cows with and without hyperketonemia

Variables	Groups		“*p*” level
	Control (*n* = 23)	Hyperketonemia (*n* = 15)	
Leptin Expression	2,17 ± 0,26^B^	3,72 ± 0,36^A^	0,0174
ObRb Expression	0,28 ± 0,23^B^	3,05 ± 0,43^A^	<,0001
Adiponectin Expression	3,42 ± 0,29	3,09 ± 0,32	0,5400
AdipoR1 Expression	4,52 ± 0,28^A^	1,83 ± 0,33^B^	<,0001
Resistin Expression	2,47 ± 0,28^A^	2,06 ± 0,20^B^	0,0435

*Different letters indicate differences at the 5% probability level.

### Multivariate analysis of mRNA gene expression of adipokines in placental tissue with biochemical variables from cows with and without hyperketonemia

**Fig. 1:**
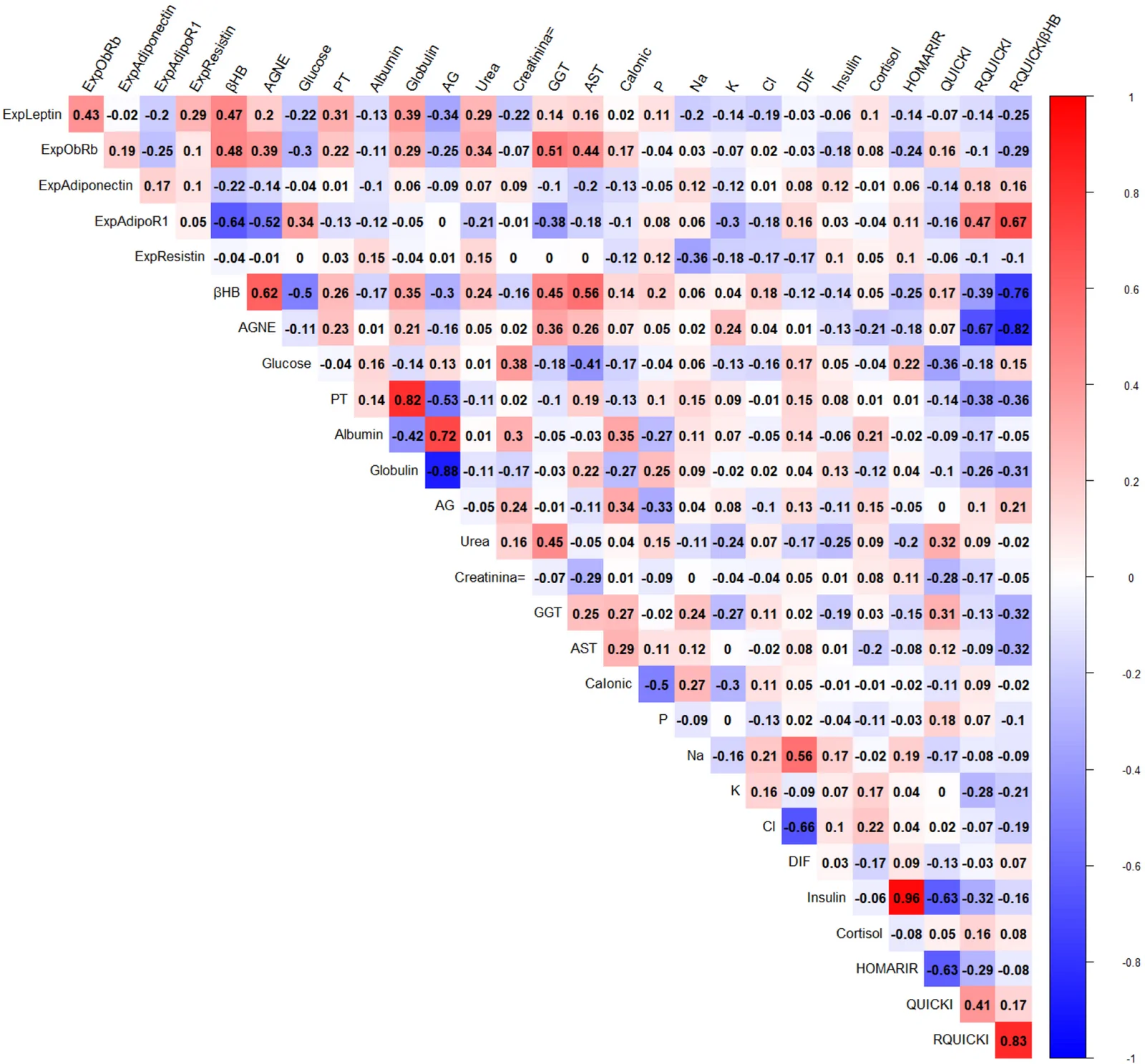
Graphical representation (Heatmap) indicating behavioral patterns of different biomarkers of the blood metabolic profile and adipokine gene expression in the placenta in cows from the control group (*n* = 23) and cows with hyperketonemia (*n* = 15) at the time of calving, where warm tones (red) indicate a high degree of correlation and cool tones (blue) indicate a low degree of correlation between pairs of variables

In the analysis of the relationship varying degrees of relationships were found. The Heatmap of the correlation between placental gene expression variables of mRNA for adipokines with different biomarkers of the metabolic profile, in cows with and without hyperketonemia, is shown in Fig. 1.

## Discussion

All cows participating in this study were in a state of high negative energy balance (NEB), confirmed by increased serum NEFA concentrations in both groups, especially in females in the hyperketosis group. Elevations in the concentrations of these metabolites, especially β-hydroxybutyrate, occur as a result of alterations in energy metabolism originating from NEB, which occur mainly in ruminant females during the transition period (Duffield et al. 2009; McArt et al. 2013). It is unclear whether the pathways that stimulate hyperketosis have been fully elucidated, a fact confirmed by the observed correlations between gene expression for adipokines and biochemical markers, which infer the dynamics of metabolic homeostasis and the physiological performance of females.

In the current study, there was a significant difference in the gene expression of mRNA for leptin and its receptor (ObRb) in the placental tissue between the groups analyzed, with an increase in expression in hyperketonemic cows. The increase in this specific group indicates that there is an adaptive response in the metabolic profile caused by this hormone. Leptin plays a fundamental role in modulating energy metabolism and its increase has been reported during negative energy balance regulation processes (Pérez-Pérez et al. 2015; D’Souza et al. 2017; Shen et al. 2020a).

The increased expression of ObRb in the placentas from cows with hyperketonemia highlights the high local influence of this adipokine in these females, when compared to females without the metabolic disorder. Studies show that placental leptin participates in the implantation, development, and growth of the embryo, in addition to actions in the placental tissue itself, including angiogenesis, immunomodulation, regulation of apopitotic processes, and growth, influencing the weight of the fetus at birth (Henson and Castracane 2006; Uzelac et al. 2010; Reverchon et al. 2014; Pérez-Pérez et al. 2015; Monroy et al. 2017; Manoharan et al. 2019; Shen et al. 2020). Furthermore, in females diagnosed with gestational diabetes, increases in local leptin levels are associated with greater nutrient absorption and glucose uptake in the placenta due to positive regulation of the expression and activity of membrane glucose transporters (GLUT1 and 3), which provide greater substrate for gluconeogenesis, increasing the expression of aquaglyceroporin membrane channels and the uptake of amino acids, and contributing to fetal hyperglycemia and systemic repercussions for the mother (Pérez-Pérez et al. 2013, 2018; Vilariño-Garcıa et al. 2018; Balachandiran et al. 2021; Guadix et al. 2023).

Additionally, a moderate positive correlation was observed between leptin gene expression and β-hydroxybutyrate as well as ObRb expression. Likewise, ObRb expression showed moderate positive correlations with β-hydroxybutyrate, NEFA, GGT, AST, and urea, and a moderate negative correlation with glucose. This set of associations suggests that activation of the leptin–ObRb axis parallels the metabolic state typical of negative energy balance, characterized by intense lipid mobilization, increased fatty acid oxidation, and greater production of ketone bodies. Thus, the data indicate that leptin signaling is integrated into the metabolic adaptations that support energy demands during this period.

Mechanistically, leptin plays a relevant role in the regulation of fatty acid oxidation. This effect occurs, in part, through the negative regulation of acetyl-CoA carboxylase (ACC) expression, reducing malonyl-CoA formation and, consequently, relieving the inhibition of carnitine palmitoyltransferase-1 (CPT1), thereby facilitating the entry of fatty acids into mitochondria for β-oxidation. In parallel, leptin-induced activation of AMP-activated protein kinase (AMPK) reinforces this pathway by also inhibiting ACC. In addition, leptin reduces hepatic expression of stearoyl-CoA desaturase-1 (SCD-1), limiting lipogenesis and re-esterification. These mechanisms, widely described in the literature (Cohen et al. 2002; Minokoshi et al. 2002; Cohen and Friedman 2004; Chilliard et al. 2005; Buettner et al. 2008; Park and Ahima 2015; Pinho et al. 2022), support the observed association between ObRb, NEFA, and β-hydroxybutyrate.

The positive correlation between ObRb and NEFA can be interpreted as a direct reflection of increased circulating fatty acid availability, while the association with β-hydroxybutyrate reinforces its link to enhanced hepatic ketogenesis. These interpretations are consistent with established metabolic mechanisms and are therefore well supported by the data. In contrast, the associations with GGT and AST suggest a possible relationship between leptin signaling and hepatic functional status, potentially mediated by increased metabolic load resulting from intensified lipid oxidation. However, this link remains more speculative, as these enzymes are influenced by multiple processes, including steatosis, inflammation, and oxidative stress. The negative correlation between ObRb and glucose may indicate a role of leptin in modulating glucose metabolism; however, within the transition period, it may also reflect broader physiological adaptations related to energy prioritization. In summary, the data consistently support an association between leptin signaling and its receptor with enhanced lipid mobilization and oxidation, whereas their potential implications for hepatic function and hyperketonemia, although plausible, still require further investigation to establish more robust causal relationships.

In this sense, the pathways activated by leptin can consequently increase ketogenesis, resulting in increase in ketone bodies, reinforcing the modulating role of this hormone in energy metabolism. Simultaneously, as a result of these alterations and repercussions on hepatic metabolism, the levels of variables signaling injury to this organ may undergo changes, as observed in the current study through the significant increase in serum GGT activity in the group of hyperketonemic cows in relation to the control group, which may indicate the presence of a certain degree of hepatocellular impairment, as well as a moderate correlation between β-hydroxybutyrate and this enzyme. Despite not being a hepatospecific enzyme, when elevated, leptin is commonly associated with cases of increased hepatic metabolism, as occurs in the transition period, or even in processes that lead to liver damage, such as fatty infiltration triggered by exacerbated lipomobilization in cases of hyperketonemia, which justifies the findings (Kaneko et al. 2008; González and Silva 2017; Souto et al. 2019; Mohsin et al. 2022). Regarding serum AST activity, there was no difference between the groups, however the moderate positive correlations with β-hydroxybutyrate and ObRb gene expression are analogous to GGT in relation to the associated metabolic processes.

Another significantly different variable between the groups was urea, with higher concentrations in hyperketonemic cows. Urea is a product of protein metabolism and amino acid catabolism in the liver and its concentrations in the blood can be influenced by various metabolic processes. Therefore, this increase may be associated with multiple factors in the metabolism of these animals, including the regulation of the metabolism of proteins for energy (González and Silva 2017). The moderate positive correlation of this variable with the expression of ObRb suggests that the metabolic pathways associated with leptin and protein catabolism may be interconnected and/or occur simultaneously, since both aim to increase energy availability to the body.

Although no difference was identified in the expression of adiponectin between the groups, a significant reduction in the expression of its receptor, AdipoR1, was found in placentas from hyperketonemic cows, indicating that the modulating action of this adipokine during pregnancy is restricted when the metabolic disorder is present, or even that the reduction in the actions of this adipokine can contribute to the systemic condition. Adiponectin is known to improve insulin sensitivity by regulating the biological actions of growth factors and calcium influx by activating calcium/calmodulin-dependent protein kinase (CaMK) and adenosine monophosphate-activated protein kinase (AMPK), which also promotes regulation of fatty acid oxidation and glucose metabolism; in the placenta, this adipokine modulates the transport of nutrients, influences placentogenesis, and regulates the transport of glucose and triglycerides, contributing positively to the development and increase in fetal weight and fetal and maternal health (Yamauchi et al. 2003, 2014; Kakizaki et al. 2008; Luo and Liu 2016; Roh et al. 2016; Shen et al. 2020a).

Therefore, expression values for AdipoR1 in placentas from healthy cows may indicate a response to adequate metabolic status. Negative correlations between AdipoR1 gene expression with β-hydroxybutyrate (high), NEFA (moderate), and serum GGT activity (moderate) were also observed and can be justified by the reduction in adiponectin concentration in conditions of chronic inflammation and metabolic diseases, such as hyperketonemia, as well as during pregnancy (Polyzos et al. 2010; Godoy-Matos et al. 2014; Kesser et al. 2015; Kasimanickam 2016; Luo and Liu 2016; Mann et al. 2018). Furthermore, the decrease in levels of this hormone in cows with type II ketosis was associated with the presence of fatty liver and the formation of insulin resistance in peripheral tissues, due to the reduction in the activity of its intracellular receptor (Shen et al. 2020b).

A moderate positive correlation was found between AdipoR1 and glucose, consistent with the glycemic modulation actions caused by this adipokine (Yamauchi et al. 2003, 2014; Luo and Liu 2016; Roh et al. 2016). Furthermore, a high negative correlation of glucose with β-hydroxybutyrate was observed, which can be explained by the physiological mechanisms underlying the regulation of energy metabolism, especially in NEB conditions. In the current study, both groups presented hyperglycemia, however, it is noteworthy that the mean glucose concentrations were significantly higher in the control group of cows. A similar condition was found in cows with NEB during the transition period by Hajimohammadi and Chalmeh (2016) and in hyperketonemic cows by Soares et al. (2019). However, it is important to emphasize that all the females studied here were going through the calving event when the blood samples were collected, which is naturally an episode of great stress, confirmed by the increase in serum cortisol concentrations in both groups, which possibly influenced the glycemic status of these animals (Kaneko et al. 2008; Simonov and Vlizlo 2013).

Another factor to be considered is the ability to capture and use glucose in tissues in a manner dependent on the presence of insulin. Here we found that cows with hyperketonemia had a significantly lower mean RQUICKI_BHB_ value when compared to the control group, demonstrating lower sensitivity and greater tissue resistance to insulin. The RQUICKI and RQUICKI_BHB_ indices were also strongly negatively correlated with the biochemical variables of the energy profile, β-hydroxybutyrate and NEFA, and moderately with the serum activity of GGT, which is justified by the impairment of the biological efficacy of insulin in cows with prolonged hyperketonemia (Kerestes et al. 2009; Djoković et al. 2017; Soares et al. 2019). The action of insulin can also be influenced by the actions of other hormones, such as leptin, either by its direct action on pancreatic β-cells, reducing the expression, biosynthesis, and secretion of the hormone, or by its ability to reduce insulin sensitivity in target tissues, which is probably associated with increased oxidation of fatty acids, modulation of insulin receptors, by interference with cell signaling pathways, or even by its collaboration in the systemic inflammatory state during metabolic disorder (Chilliard et al. 2005; Guilherme et al. 2008; Marroqui et al. 2012; Al-Badri et al. 2015; Pérez-Pérez et al. 2016; Bungau et al. 2020; Shen et al. 2020b; Guadix et al. 2023).

Furthermore, insulin sensitivity in peripheral tissues is reduced under conditions of reduced adiponectin action (Shen et al. 2020b), as found in the hyperketonemic cows in the current study. Additionally, a high positive correlation between AdipoR1 gene expression and RQUICKI_BHB_ and moderate positive correlation with RQUICKI was found. Shen et al. (2020b), when studying serum adipokines in cows and their roles in type I and II ketosis, observed that the RQUICKI index correlated significantly positively with adiponectin and associated this finding with the insulin sensitivity stimulatory capacity of this adipokine. Therefore, the positive correlation of the adiponectin receptor with the RQUICKI and RQUICKI_BHB_ indices indicates that the active presence of adiponectin is associated with a metabolic response that favors insulin sensitivity and, therefore, influences energy homeostasis.

Higher means of resistin expression in placentas of cows without hyperketonemia were evidenced, which may indicate a positive influence of resistin’s action on the energy metabolism in these females, promoting better glycemic control, or that its reduction could contribute to energy imbalance in hyperketonemic cows. Studies have demystified the correlations between resistin and insulin resistance. Tsiotra et al. (2008), Kuzmicki et al. (2009), and Gharibeh et al. (2010) showed that patients with DM or GDM present greater expression of this adipokine when compared to healthy patients, including increased expression in peripheral blood mononuclear cells. Other studies show that resistin participates in the induction of insulin resistance through its expression in hepatocytes or through the inflammatory pathways in which this adipokine is involved (Sheng et al. 2008; Kuzmicki et al. 2009). However, Shen et al. (2020b) did not observe a correlation between the insulin sensitivity check index (RQUICKI) and this adipokine in cows with ketosis, as in the current study.

In conclusion, the analysis of mRNA expression of adipokines produced in the placenta was shown to be a novel tool to understand the role of these molecules in the metabolism regulation mechanisms in ruminant females, since, as observed in the current study, the interaction of these variables with the metabolites of cows contributes to the understanding of the dynamics of hyperketonemia. Of note were the increased gene expression of leptin, and its receptor (ObRb), and the reduced gene expression of the adiponectin receptor (AdipoR1) in cows with the metabolic disorder studied. Therefore, it can be concluded that the placental production of adipokines makes an important contribution to the metabolic conditions found in cows during the transition period, having implications for the physiology, health, and productivity of females.

Despite the consistency of the observed associations, it is important to consider certain limitations of the study. The relatively small number of samples used for placental gene expression analysis, combined with the limited size of the hyperketonemic group (*n* = 15), may have made it more difficult to detect more subtle relationships among variables and contributed to greater data variability. This scenario may, consequently, affect comparisons between groups and increase the likelihood of sampling bias. In addition, the influence of factors such as breed and specific farm management conditions (including diet, production system, and environment) cannot be completely ruled out, as they may have interfered with the observed results. These aspects call for caution when generalizing the findings, since different production contexts may exhibit distinct metabolic responses.

## Data Availability

The data supporting the conclusions of this study are available in the article and its Supplementary Information. However, access to all spreadsheets, as well as individual animal data that corroborate the conclusions of this study, is not publicly available due to confidentiality issues with other associated projects and can be obtained upon reasonable request to the corresponding author.

## Footnotes

1 DEPC Water™, Thermo Fisher Scientific, United States of America.

2 β-hidroxibutirato /Rambut, RANDOX Laboratories Ltd./ref.RB1008, Reino Unido.

3 NEFA, RANDOX Laboratories Ltd./ref.FA115, Reino Unido.

4 Glucose Liquiform, Labtest Diagnostics S.A., Brazil.

5 Total protein, Labtest Diagnostics S.A., Brazil.

6 Albumin, Labtest Diagnostics S.A., Brazil.

7 Urea, Labtest Diagnostics S.A., Brazil.

8 Creatinine, Labtest Diagnostics S.A., Brazil.

9 Gamma GT Liquiform, Labtest Diagnostics S.A., Brazil.

10 AST/GOT Liquiform, Labtest Diagnostics S.A., Brazil.

11 Chlorides Liquiform, Labtest Diagnostics S.A., Brazil.

12 Phosphorus UV Liquiform, Labtest Diagnostics S.A., Brazil.

13 Access Immunoassay, Beckman Coulter, United States of America.

14 Access Immunoassay, Beckman Coulter, United States of America.

## References

[CR1] Acquarone E, Monacelli F, Borghi R et al (2019) Resistin: A reappraisal. Mech Ageing Dev 178:46–63. 10.1016/j.mad.2019.01.004 30650338

[CR2] Al-Badri MR, Zantout MS, Azar ST (2015) The role of adipokines in gestational diabetes mellitus. Ther Adv Endocrinol Metab 6:103–108. 10.1177/2042018815577039 PMC4480551 26137214

[CR3] Balachandiran M, Bobby Z, Dorairajan G et al (2021) Decreased maternal serum adiponectin and increased insulin-like growth factor-1 levels along with increased placental glucose transporter-1 expression in gestational diabetes mellitus: Possible role in fetal overgrowth. Placenta 104:71–80. 10.1016/j.placenta.2020.11.008 33285436

[CR4] Buettner C, Muse ED, Cheng A et al (2008) Leptin controls adipose tissue lipogenesis via central, stat3-independent mechanisms. Nat Med 14:667–675. 10.1038/nm1775 PMC2671848 18516053

[CR5] Bungau S, Behl T, Tit DM et al (2020) Interactions between leptin and insulin resistance in patients with prediabetes, with and without NAFLD. Exp Ther Med 20:197. 10.3892/etm.2020.9327 PMC7588790 33123227

[CR6] Chilliard Y, Delavaud C, Bonnet M (2005) Leptin expression in ruminants: nutritional and physiological regulations in relation with energy metabolism. Domest Anim Endocrinol 29:3–22. 10.1016/j.domaniend.2005.02.026 15876510

[CR7] Cohen P, Friedman JM (2004) Leptin and the control of metabolism: role of stearoyl-coA desaturase-1 (SDC-1). Nutr J 134:2455S–63S. 10.1093/jn/134.9.2455S 15333742

[CR8] Cohen P, Miyazaki M, Socci ND et al (2002) Role for stearoyl-CoA desaturase-1 in leptin-mediated weight loss. Sci Webinars 297:240–243. 10.1126/science.1071527 12114623

[CR9] Constable PD, Stämpfli HR (2005) Experimental determination of net protein charge and Atot and Ka of nonvolatile buffers in canine plasma. J Vet Intern Med 19:507–514 16095167

[CR12] D’Souza AM, Neumann UH, Glavas MM et al (2017) The glucoregulatory actions of leptin. Mol Metab 6:1052–1065. 10.1016/j.molmet.2017.04.011 PMC5605734 28951828

[CR10] Dirksen G, Gründer HD, Stôber M (1993) Rosenberger: Exame Clínico dos Bovinos. Ed. 3ª. Guanabara Koogan, Rio de Janeiro, p 419

[CR11] Djoković R, Dosković V, Cincović M et al (2017) Estimation of Insulin Resistance in Healthy and Ketotic Cows during an Intravenous Glucose Tolerance Test. Pak Vet J 37:387–392

[CR67] Duarte SS, Gomes-Filho MA, Silva DMF et al (2014) Expressão gênica de adipocinas em ovelhas alimentadas com resíduos da indústria do biodiesel da mamona. Arq Bras Med Vet Zootec 66:1171–1178. 10.1590/1678-6355

[CR13] Duffield TF, Lissemore KD, McBride BW et al (2009) Impact of hyperketonemia in early lactation dairy cows on health and production. J dairy sci 92:571–580. 10.3168/jds.2008-1507 19164667

[CR14] Edmonson AJ, Magro IJ, Tecelão LD et al (1989) A body condition scoring chart for Holstein dairy cows. J dairy sci 72:68–78. 10.3168/jds.S0022-0302(89)79081-0

[CR15] Gharibeh MY, Al Tawallbeh GM, Abboud MM et al (2010) Correlation of plasma resistin with obesity and insulin resistance in type 2 diabetic patients. Diabetes Metab J 36:443–449. 10.1016/j.diabet.2010.05.003 20739208

[CR16] Godoy-Matos A, Cruz I, Costa R et al (2014) Adipocinas: uma visão geral dos seus efeitos metabólicos. Rev Hosp Univ Pedro Ernesto 13:54–60. 10.12957/rhupe.2014.9806

[CR17] González FHD, Silva SC (2017) Introdução à bioquímica clínica veterinária. Ed. 3º. UFRGS, Porto Alegre, p 535

[CR18] Gordon JL, Leblanc SJ, Duffield TF (2013) Ketosis treatment in lactating dairy cattle. Vet Clin North Am Food Anim Pract 29:433–445. 10.1016/j.cvfa.2013.03.001 23809899

[CR19] Guadix P, Corrales I, Vilariño-García T et al (2023) Expression of nutrient transporters in placentas affected by gestational diabetes: role of leptin. Front Endocrinol 14:1172831. 10.3389/fendo.2023.1172831 PMC10366688 37497352

[CR20] Guilherme A, Virbasius JV, Puri V et al (2008) Adipocyte dysfunctions linking obesity to insulin resistance and type 2 diabetes. Nat Rev Mol Cell Biol 9:367–377. 10.1038/nrm2391 PMC2886982 18401346

[CR21] Hajimohammadi A, Chalmeh AA (2016) Circulating metabolic hormones in different metabolic states of high producing Holstein dairy cows. Iran J Vet Med 10:277–284. 10.22059/IJVM.2016.59721

[CR22] Henson MC, Castracane VD (2006) Leptin in pregnancy: an update. Biol Reprod 74:218–229. 10.1095/biolreprod.105.045120 16267210

[CR23] Holtenius P, Holtenius K (2007) A model to estimate insulin sensitivity in dairy cows. Acta Vet Scand 49:1–3. 10.1186/1751-0147-49-29 PMC2092429 17931417

[CR24] Kakizaki S, Sohara N, Yamazaki Y et al (2008) Elevated plasma resistin concentrations in patients with liver cirrhosis. J Gastroenterol Hepatol 23:73–77. 10.1111/j.1440-1746.2006.04757.x 18171344

[CR25] Kaneko JJ, Harvey JW, Bruss ML (2008) Clinical Biochemistry of Domestic Animals, 6th edn. Academic, San Diego, p 916

[CR26] Kasimanickam RK (2016) Subclinical pregnancy toxemia-induced gene expression changes in ovine placenta and uterus. Front Vet Sci 3:69. 10.3389/fvets.2016.00069 PMC5003868 27626035

[CR27] Kerestes M, Faigl V, Kulcsár M et al (2009) Periparturient insulin secretion and whole-body insulin responsiveness in dairy cows showing various forms of ketone pattern with or without puerperal metritis. Domest Anim Endocrinol 37:250–261. 10.1016/j.domaniend.2009.07.003 19716674

[CR28] Kesser J, Colina M, Heinz JFL et al (2015) The rapid increase of circulating adiponectin in neonatal calves depends on colostrum intake. J dairy sci 98:7044–7051. 10.3168/jds.2015-9726 26277307

[CR29] Kuzmicki M, Telejko B, Szamatowicz J et al (2009) High resistin and interleukin-6 levels are associated with gestational diabetes mellitus. Gynecol Endocrinol 25:258–263. 10.1080/09513590802653825 19408175

[CR30] Leblanc S (2010) Monitoring metabolic health of dairy cattle in the transition period. J Reprod Dev 56:S29–S35. 10.1262/jrd.1056S29 20629214

[CR31] Little TM, Hills FJ (1978) Agricultural experimentation: design and analysis. Wiley, New York, p 350

[CR32] Livak KJ, Schmittgen TD (2001) Analysis of relative gene expression data using real-time quantitative PCR and the 2 – ∆∆CT method. Meth 25:402–408. 10.1006/meth.2001.1262 11846609

[CR33] Loiklung C, Sukon P, Thamrongyoswittayakul C (2022) Global prevalence of subclinical ketosis in dairy cows: A systematic review and meta-analysis. Res Vet Sci 144:66–76. 10.1016/j.rvsc.2022.01.003 35077992

[CR34] Luo L, Liu M (2016) Adipose tissue in control of metabolism. J Endocrinol 231:R77–R99. 10.1530/JOE-16-0211 PMC7928204 27935822

[CR35] Mann S, Urh C, Sauerwein H et al (2018) Short communication: The association of adiponectin and leptin concentrations with prepartum dietary energy supply, parity, body condition, and postpartum hyperketonemia in transition dairy cows. J dairy sci 101:806–811. 10.3168/jds.2017-13752 29103711

[CR36] Manoharan B, Bobby Z, Dorairajan G et al (2019) Adipokine levels and their association with insulin resistance and fetal outcomes among the newborns of Indian gestational diabetic mothers. Saudi Med J 40:353–359. 10.15537/smj.2019.4.24058 PMC6506657 30957128

[CR37] Marroqui L, Gonzalez A, Ñeco P et al (2012) Role of leptin in the pancreatic beta-cell: effects and signaling pathways. J Mol Endocrinol 49:R9–R17. 10.1530/JME-12-0025 22448029

[CR38] Masevicius FD, Vazquez AR, Enrico C et al (2013) Diferença de íons fortes na urina como determinante importante das alterações na concentração plasmática de cloreto em pacientes no pós-operatório. Rev Bras Ter Intensiva 25:197–204. 10.5935/0103-507X.20130035 PMC4031852 24213082

[CR39] McART JAA, Nydam DV, Oetzel GR et al (2013) Elevated non-esterified fatty acids and β-hydroxybutyrate and their association with transition dairy cow performance. Vet J 198:560–570. 10.1016/j.tvjl.2013.08.011 24054909

[CR40] Minokoshi Y, Kim YB, Peroni OD et al (2002) Leptin stimulates fatty-acid oxidation by activating amp-activated protein kinase. Nat 415:339–343. 10.1038/415339 11797013

[CR41] Mohsin MA, Yu H, He R et al (2022) Differentiation of subclinical ketosis and liver function test indices in adipose tissues associated with hyperketonemia in postpartum dairy cattle. Front Vet Sci 8:796494. 10.3389/fvets.2021.796494 PMC8850981 35187139

[CR42] Monroy MLLLV, González-Domínguez MI, Zaina S et al (2017) Leptin and its Receptors in Human Placenta of Small, Adequate, and Large for Gestational Age Newborns. Horm Metab Res 49:350–358. 10.1055/s-0043-103345 28351089

[CR43] Park K, Ahima RS (2015) Physiology of leptin: energy homeostasis, neuroendocrine function and metabolism. Metabol 64:24–34. 10.1016/j.metabol.2014.08.004 PMC4267898 25199978

[CR44] Pérez-Pérez A, Maymó JL, Gambino YP et al (2013) Activated translation signaling in placenta from pregnant women with gestational diabetes mellitus: possible role of leptin. Horm Metab Res 45:436–442. 10.1055/S-0032-1333276 23386416

[CR47] Pérez-Pérez A, Sánchez-Jiménez F, Maymó J et al (2015) Role of leptin in female reproduction. Clin Chem Lab Med 53:15–28. 10.1515/cclm-2014-0387 25014521

[CR45] Pérez-Pérez A, Guadix P, Maymó J et al (2016) Insulin and leptin signaling in placenta from gestational diabetic subjects. Horm Metab Res 48:62–69. 10.1055/S-0035-1559722 26584065

[CR46] Pérez-Pérez A, Toro A, Vilariño-García T et al (2018) Leptin action in normal and pathological pregnancies. J Cel Mol Med 22:716–727. 10.1111/JCMM.13369 PMC5783877 29160594

[CR48] Perseghin G, Caumo A, Caloni M et al (2001) Incorporation of the fasting plasma FFA concentration into QUICKI improves its association with insulin sensitivity in nonobese individuals. J Clin Endocrinol Metabol 86:4776–4781. 10.1210/jcem.86.10.7902 11600540

[CR49] Pinho KHS, Paiva MJ, Oliveira RAC (2022) Leptina e adiponectina: papel dos hormônios nos processos metabólicos e impactos na sua desregulação. Res Soc Dev 11:e34711225144. 10.33448/rsd-v11i2.25144

[CR50] Polyzos SA, Kountouras J, Zavos C et al (2010) The role of adiponectin in the pathogenesis and treatment of non-alcoholic fatty liver disease. Diabetes Obes Metab 12:365–383. 10.1111/j.1463-1326.2009.01176.x 20415685

[CR51] Reverchon M, Ramé C, Bertoldo M et al (2014) Adipokines and the female reproductive tract. Int J Endocrinol 2014:232454. 10.1155/2014/232454 PMC3948585 24695544

[CR52] Roh SG, Suzuki Y, Gotoh T et al (2016) Physiological roles of adipokines, hepatokines, and myokines in ruminants. Asian-Australas J Anim Sci 29:1–15. 10.5713/ajas.16.0001R PMC4698675 26732322

[CR59] Statistical Analyses Sistem Institute (2000) Inc 1996. SAS user´s guide: Statics Version. SAS, Cary, N.C.

[CR53] Shen L, Zhu Y, Xiao J et al (2020a) Relationships between placental adiponectin, leptin, visfatin and resistin and birthweight in cattle. Reprod Fertil Devel 32:402–408. 10.1071/RD18247 31739842

[CR54] Shen L, Zhu Y, Xiao J et al (2020b) Serum adipokines play different roles in type I and II ketosis. Asian-Australasian J Anim Sci 33:1930–1939. 10.5713/ajas.19.0728 PMC7649395 32054179

[CR55] Sheng CH, Di J, Jin Y et al (2008) Resistin is expressed in human hepatocytes and induces insulin resistance. End 33:35–143. 10.1007/s12020-008-9065-y 18446452

[CR56] Simonov MR, Vlizlo VV (2013) Some indicators of protein metabolism in blood of cows under ketosis. Anim Biol 15:120–124. 10.15407/animbiol15.03.120

[CR57] Soares GSL, Ribeiro ACS, Cajueiro JFP et al (2019) Cardiac biomarkers and blood metabolites in cows with clinical ketosis. Semin Ciên Agrár 40:3525–3539. 10.5433/1679-0359.2019v40n6Supl3p3525

[CR58] Souto RCJ, Afonso JAB, Mendonça CL et al (2019) Biochemical, endocrine, and histopathological profile of liver and kidneys of sheep with pregnancy toxemia. Pesq Vet Bras 39:780–788. 10.1590/1678-5150-PVB-6400

[CR60] Tatone EH, Gordon JL, Hubbs J et al (2016) A systematic review and meta-analysis of the diagnostic accuracy of point-of-care tests for the detection of hyperketonemia in dairy cows. Prev Vet Med 130:18–32. 10.1016/j.prevetmed.2016.06.002 27435643

[CR61] Tatone EH, Duffield TF, LeBlanc SJ et al (2017) Investigating the within-herd prevalence and risk factors for ketosis in dairy cattle in Ontario as diagnosed by the test-day concentration of β-hydroxybutyrate in milk. J Dairy Sci 100:1308–1318. 10.3168/jds.2016-11453 28012623

[CR62] Tsiotra PC, Tsigos C, Anastasiou E et al (2008) Peripheral mononuclear cell resistin mRNA expression is increased in type 2 diabetic women. Mediat Inflamm 2008:892864. 10.1155/2008/892864 PMC2606019 19125180

[CR63] Uzelac PS, Li X, Lin J et al (2010) Dysregulation of leptin and testosterone production and their receptor expression in the human placenta with gestational diabetes mellitus. Placenta 31:581–588. 10.1016/j.placenta.2010.04.002 20421132

[CR64] Vilariño-García T, Pérez-Pérez A, Dietrich V et al (2018) Leptin upregulates aquaporin 9 expression in human placenta in vitro. Gynecol Endocrinol 34:175–177. 10.1080/09513590.2017.1380184 28942694

[CR66] Yamauchi T, Kamon J, Ito Y et al (2003) Cloning of adiponectin receptors that mediate antidiabetic metabolic effects. Nat 423:762–769 10.1038/nature01705 12802337

[CR65] Yamauchi T, Iwabu M, Okada-Iwabu M et al (2014) Adiponectin receptors: A review of their structure, function and how they work. Best Pract Res Clin Endocrinol Metab 28:15–23. 10.1016/j.beem.2013.09.003 24417942

